# Characterization of a conditional interleukin‐1 receptor 1 mouse mutant using the Cre/LoxP system

**DOI:** 10.1002/eji.201546075

**Published:** 2016-01-18

**Authors:** Wesam H. Abdulaal, Catherine R. Walker, Ryan Costello, Elena Redondo‐Castro, Ilgiz A. Mufazalov, Athina Papaemmanouil, Nancy J. Rothwell, Stuart M. Allan, Ari Waisman, Emmanuel Pinteaux, Werner Müller

**Affiliations:** ^1^ Faculty of Life SciencesUniversity of ManchesterManchesterUK; ^2^ Biochemistry Department, Faculty of SciencesKing Abdulaziz UniversityJeddahKingdom of Saudi Arabia; ^3^ Institute for Molecular MedicineMedical University of Johannes Gutenberg‐University of MainzMainzGermany

**Keywords:** Cre/loxP, IL‐17, Infection, Immune regulation, *Trichuris muris*

## Abstract

IL‐1 is a key cytokine known to drive chronic inflammation and to regulate many physiological, immunological, and neuroimmunological responses via actions on diverse cell types of the body. To determine the mechanisms of IL‐1 actions as part of the inflammatory response in vivo, we generated a conditional IL‐1 receptor 1 (IL‐1R1) mouse mutant using the Cre/LoxP system (IL‐1R1^fl/fl^). In the mutant generated, exon 5, which encodes part of the extracellular‐binding region of the receptor, is flanked by LoxP sites, thereby inactivating the two previously described functional IL‐1R1 gene transcripts after Cre‐mediated recombination. Using keratin 14‐Cre driver mice, new IL‐1R1 deficient (−/−) mice were subsequently generated, in which all signaling IL‐1 receptor isoforms are deleted ubiquitously. Furthermore, using vav‐iCre driver mice, we deleted IL‐1 receptor isoforms in the hematopoietic system. In these mice, we show that both the IL‐17 and IL‐22 cytokine response is reduced, when mice are challenged by the helminth *Trichuris muris*. We are currently crossing IL‐1R1^fl/fl^ mice with different Cre‐expressing mice in order to study mechanisms of acute and chronic inflammatory diseases.

## Introduction

IL‐1 is a key driver of inflammation in many acute and chronic inflammatory disorders, including infection, stroke, inflammatory bowel disease, type 2 diabetes, and multiple sclerosis. IL‐1 exists as two agonists (IL‐1α and IL‐1β) that bind to the main signaling IL‐1 type 1 receptor (IL‐1R1) leading to amplification of the inflammatory response. So far, the field of inflammation has used IL‐1R1^−/−^ mice originally generated by targeted deletion of exon 1 and 2 of the *il1r1* gene 1, 2, showing that most IL‐1 actions are mediated by IL‐1R1. However, studies using these mice with helminth (*Trichuris muris*) infection 3 and stroke 4 found that IL‐1 can function in an IL‐1R1‐independent manner, pointing to a residual IL‐1 signaling capacity in the classical IL‐1R1^−/−^ mice. A recent study found that an additional internal promoter adjacent to exon 3 leads to the expression of IL‐1R3, a truncated isoform of the receptor to which IL‐1 is still capable of binding, accounting for IL‐1R1‐independent IL‐1 actions seen in the classical IL‐1R1^−/−^ mice 5. Therefore, there is a need for an improved mouse mutant in which all signaling IL‐1 receptor isoforms are genetically deleted.

Although many mechanisms of IL‐1 actions in inflammatory disorders have been identified, two important unanswered questions in the field of IL‐1 biology remain, i.e. which cells produce and which cells respond to IL‐1 ligands physiologically and in different disease states. To this end, the Cre/loxP system aimed at selective/conditional genetic deletion remains the best experimental approach to answer such questions 6. We have generated a new conditional IL‐1R1 mouse mutant, named IL‐1R1 conditional allele (IL‐1R1^fl/fl^) in which exon 5, flanked with LoxP sites, is deleted selectively under cell/tissue‐specific Cre recombinase expression. Deletion of exon 5 eliminates part of the extracellular region of the *il1r1* gene generating a frameshift from exon 4 to all downstream exons and, therefore, disrupting all internal promoter sites and leading to genetic inhibition of all IL‐1 receptor isoforms.

We describe here the generation of a new ubiquitous IL‐1R1^−/−^ line obtained by crossing IL‐1R1^fl/fl^ mice with mice expressing Cre recombinase under the control of the human keratin 14 promoter 7. As a first step toward identifying the target cell on which IL‐1 acts during infection, we have generated a mouse line in which all IL‐1 receptor isoforms are deleted in hematopoetic cells by crossing IL‐1R1^fl/fl^ mice with mice expressing Cre recombinase under the control of the vav promoter 8, 9. In both mutants, we show that IL‐1‐specific responses are completely abolished after conditional deletion of exon 5. Using a gut‐specific nematode infection model (low dose infection with *Trichuris muris*), we show that selective IL‐1R1 deficiency in the hematopoietic system leads to more severe pathology and reduced IL‐17 and IL‐22 responses. We conclude that the phenotypic characterization of the new IL‐1R1^fl/fl^ mouse mutant means that the allele can now be used to analyze the role of IL‐1 signaling in different cell types and in various inflammatory diseases.

## Results and discussion

### Generation of a conditional IL‐1 receptor 1‐deficient mouse mutant

**Figure 1:**
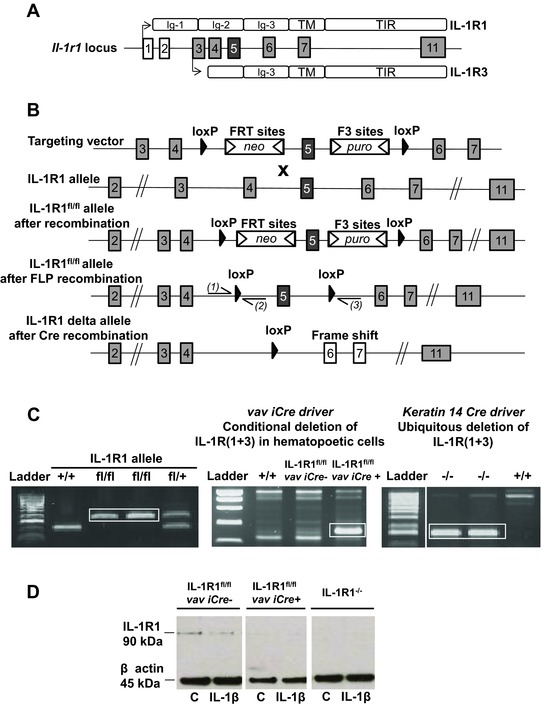
Generation of IL‐1R1^fl/fl^ mice and conditional deletion of IL‐1R1. (A) Diagram showing the *il1r1* gene locus, with each box representing exons leading to IL‐1R1 protein expression. Left arrows depict original promoter that leads to the expression of full‐length IL‐1R1, whereas arrow adjacent to exon 3 depicts an internal promoter that leads to expression of a truncated isoform of IL‐1R1 (named IL‐1R3). White boxes represent exon 1 and 2, which are targeted for the generation of original commercial IL‐1R1^−/−^ animals, in which expression of IL‐1R3 still occurs. (B) Genetic approach to generate IL‐1R1^fl/fl^ mice was designed to induce deletion of the extracellular Ig‐like C2 domain of IL‐1R1 (encoded by exon 5), generating a frameshift from exon 4 to all downstream exons leading to genetic inhibition of IL‐1R1 and IL‐1R3. (C) A representative genotyping PCR result for the identification of wild type (+/+), heterozygous (fl/+), and IL‐1R1^fl/fl^ mice, using primer pairs (1) and (2) (sequence in Methods section) is shown. Gene deletion was achieved by the excision of a loxP flanked (floxed) critical region by crossing IL‐1R1^fl/fl^ mice with mice expressing Cre recombinase under a keratin 14 promoter generating a new IL‐1R1^−/−^ in which full‐length IL‐1R1 and IL‐1R3 is deleted, or with mice expressing Cre recombinase under vav promoter, leading to deletion of all IL‐1 signaling in hematopoietic cells. Generation of the delta alleles was confirmed by genotyping PCR (bands shown by white boxes in middle and right PCR gels, using primer pairs (1) and (3) (sequence in Methods section). (D) Western blot analysis (using anti‐IL‐1R1 antibody, Abcam) from isolated spleen cells untreated or treated with IL‐1β (20 ng/mL) for 24 h showing a 90 kDa band that corresponded to IL‐1R1 in IL‐1R1^fl/fl^ vav iCre‐ mice, and lack of IL‐1R1 expression in IL‐1R1^−/−^ and IL‐1R1^fl/fl^ vav iCre+ mice. β‐Actin was used as a loading control. (C, D) Data shown are from single experiments representative of three separate experiments.

A conditional IL‐1R1 allele was generated by gene targeting in embryonic stem cells of the C57BL/6 background. The strategy of targeting and functional characterization of the allele (Fig. 1A) is depicted in Fig. 1. We positioned the loxP sites around exon 5 of the *il1r1* gene (encoding amino‐acids 166–222, which corresponds to half of the Ig‐like C2 type 2 region) that generates a frame shift when exon 5 is deleted (Fig. 1B). By positioning the loxP sites around this exon, we prevented the production of the two known transcribed functional forms of the *il1r1* gene after Cre‐mediated recombination.

PCR analysis identified the gene targeting event, and PCR product sequencing revealed the correct sequence for both the conditional loxP containing allele and the delta allele generated upon Cre‐mediated deletion (Fig. 1C). We generated two subsequent mouse mutants, one line containing the deleted allele of the *il1r1* genes (IL‐1R1^−/−^) in all tissues, and one in which the gene is deleted in the hematopoietic system (IL‐1R1^fl/fl^ vav iCre+) (Fig. 1C) (both mice showed no phenotypic alterations, not shown). IL‐1R1 protein expression was impaired by the mutation, as shown by Western blot analysis from spleen cell lysates stimulated with IL‐1β (known inducer of IL‐1 receptor expression used to fully test the functional deletion of the *il1r1* gene). We observed a complete absence of IL‐1R1 expression from spleen cells isolated from IL‐1R1^−/−^ and IL‐1R1^fl/fl^ vav iCre+ mice (Fig. 1D).

### Conditional deletion of IL‐1R1 abolishes responses to IL‐1

**Figure 2:**
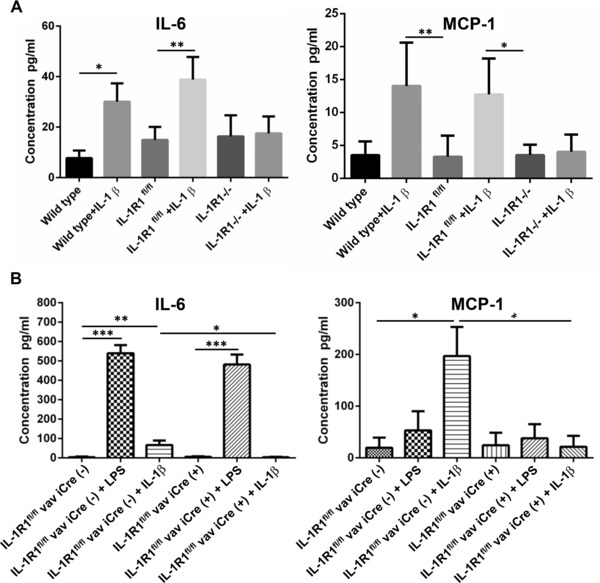
IL‐1‐induced expression of IL‐6 and MCP‐1 is abolished after conditional deletion of IL‐1R1 in isolated spleen cells. (A, B) Spleen cells isolated from (A) wild type (C57BL/6), IL‐1R1^fl/fl^, IL‐1R1^−/−^, or (B) IL‐1R1^fl/fl^ vav iCre‐ or IL‐1R1^fl/fl^ vav iCre+ mice were left untreated, or were treated with IL‐1β (20 ng/mL) or LPS (100 ng/mL) for 24 h. After 24 h, culture supernatants were collected, and levels of IL‐6 and MCP‐1 were assessed using specific ELISAs. The data are expressed as pg/mL, presented as mean ± SEM from four or six experiments carried out separately (*n* = 4, A; *n* = 6, B), and were analyzed statistically using two‐way ANOVA (A) or one‐way ANOVA (B), followed by a Tukey multiple comparison post hoc test. *p < 0.05, **p < 0.01, ***p < 0.001.

In order to test whether the IL‐1 receptor allele containing the two loxP sites is fully functional and that we have indeed functionally inactivated the *il1r1* gene in the conditional mutant, we stimulated spleen cells of wild type (C57Bl/6) mice, IL‐1R1^fl/fl^ mice or IL‐1R1^−/−^ mice (obtained by crossing IL‐1R1^fl/fl^ mice with keratin 14‐Cre driver mice) and found that IL‐1β‐induced IL‐6 and MCP‐1 release were identical in wild‐type mice compared to IL‐1R1^fl/fl^ mice, while IL‐1β‐induced IL‐6 and MCP‐1 release were completely abrogated in IL‐1R1^−/−^ mice (Fig. 2A). We then stimulated spleen cells of mice either carrying two alleles of the loxP flanked allele alone or in the presence of the vav iCre transgene (bred as littermates). We stimulated the cells with either LPS or IL‐1β, and measured release of IL‐6 and MCP‐1. Cells containing the loxP allele produced IL‐6 and MCP‐1 in the presence of IL‐1β but the cultures with cells containing the loxP allele in the presence of the vav iCre transgene exhibited no IL‐6 or MCP‐1 release (Fig. 2B). Positive controls showed that LPS stimulated cytokine secretion in the absence and the presence of the vav iCre transgene, suggesting that IL‐1 downstream signaling mechanisms were not affected by exon 5 deletion (since both IL‐1R1 and LPS receptor share identical downstream signaling mechanisms). We conclude that we functionally inactivated the *il1r1* gene in a Cre‐dependent manner. When we analyzed the lymphocyte compartment in the mutants, we did not detect any major changes in the lymphocyte population of mutants (Supporting Information Figs. 1–4).

### Interleukin‐1 response within the hematopoietic system controls the production of IL‐17 and IL‐22

In order to verify that the conditional allele is fully functional in vivo, we infected two groups of mice with the gut dwelling nematode *Trichuris muris*, one group with the conditional allele only and the second group with the conditional allele in the presence of the vav iCre transgene. Low dose infection of the mice with *Trichuris muris* leads to a Th1 and Th17 response, characterized with the production of a high level of parasite‐specific immunoglobulin G2a (IgG2a), IL‐17, and IL‐22, and subsequent chronic infection during which the mice are unable to expel the worm.

**Figure 3:**
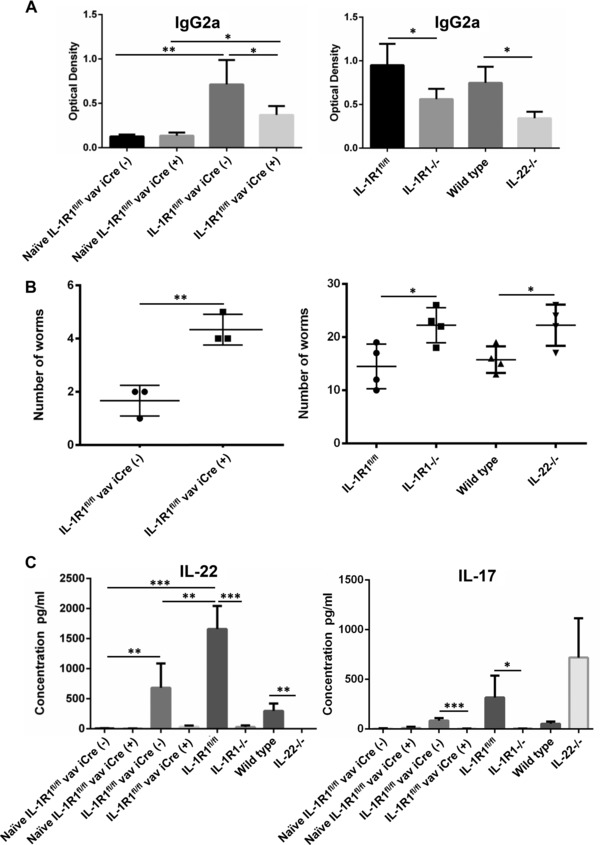
Altered immune response to Th‐1‐induced *T. muris* infection after conditional deletion of IL‐1R1. (A, B) ELISA measuring (A) specific *T. muris* IgG2 antibody in the blood and (B) worm burden counted from the colon and caecum, 21 days postinfection with low dose of *T. muris* eggs, of IL‐1R1^fl/fl^ vav iCre‐, IL‐1R1^fl/fl^ vav iCre+, IL‐1R1^fl/fl^, IL‐1R1^−/−^, IL‐22^−/−^, or wild‐type mice. (C) The blood levels of IL‐17 and IL‐22 from isolated MLN cells obtained from uninfected (naïve) or infected IL‐1R1^fl/fl^ vav iCre‐, IL‐1R1^fl/fl^ vav iCre+, IL‐1R1^fl/fl^, IL‐1R1^−/−^, IL‐22^−/−^ or wild‐type mice was determined by ELISA. The data are presented as a mean ± SEM from three or four experiments carried out separately (*n* = 4 per experimental group except for left graphs in 3A and 3B where *n* = 3 per experimental group), and were analyzed statistically using one‐way ANOVA, followed by a Tukey multiple comparison post hoc test. *p < 0.05, **p < 0.01, ***p < 0.001.

We determined the extent of the Th1 response in IL‐1R1^fl/fl^ vav iCre+ mice and show that the lack of IL‐1R1 in the hematopoietic system leads to a reduction in Th1 response, indicated by a decreased level of parasite‐specific antibodies of the IgG2a class (Fig. 3A) compared to IL‐1R1^fl/fl^ vav iCre‐ mice. Similar effects were also detected in fully deficient IL‐1R1^−/−^ mice, as well as IL‐22^−/−^ mice. Surprisingly, worm count was increased in IL‐1R1^fl/fl^ vav iCre+ mice compared to IL‐1R1^fl/fl^ vav iCre‐ mice, and similar effect was seen in IL‐1R1^−/−^ and IL‐22^−/−^ mice, suggesting that the increased worm burden could be attributed to reduction of IL‐22 (Fig. 3B). To support this, subsequently analysis of cytokine responses in the draining lymph nodes found that IL‐22 (but also IL‐17) levels were significantly reduced after *il1r1* deletion in the hematopoietic system, with similar responses being detected in IL‐1R1^−/−^ mice (Fig. 3C).

These results indicate that IL‐1 regulates IL‐17 and IL‐22 expression during Th1‐mediated immunity, in line with previous reports 10, 11, 12. We are now dissecting the specific cell type responsible for IL‐1 actions within the hematopoietic system using other Cre driver lines. When we infected IL‐1R1^fl/fl^ CD4cre mice (*N* = 5 per group) with low dose *Trichuris muris*, we could not observe a decrease in IL‐22 production in the mesenteric lymph node compared to control (133 ± 44 pg/mL IL‐22 in IL‐1R1^fl/fl^ versus 383 ± 94 pg/mL in IL‐1R1^fl/fl^ CD4cre mice), indicating that IL‐1 signaling on CD4 cells is not responsible for the reduced production of IL‐22. As expected, we confirmed the reduced IL‐17 production in the IL‐1R1^fl/fl^ CD4 cre mice (40 ± 14 pg/mL) compared to IL‐1R1^fl/fl^ mice (131 ± 17 pg/mL).

## Concluding remarks

The generation of this new conditional IL‐1R1 mutant is part of a long‐term quest to generate tools to analyze the cellular cytokine network. Given the broad expression profile of the *il1r1* gene, we expect that this mutant will provide new insights into the cellular cytokine IL‐1 network. The way the conditional allele was designed, this mutation removes both known functional receptor forms in contrast to the currently used IL‐1R1 mutant.

## Materials and methods

### Animal experiments

The generation of the IL‐1R1^fl/fl^ mice was carried out at Taconic (Cologne, Germany). The vav iCre mice were provided by Dimitris Kioussis. IL‐22^−/−^ mice were generated by Dr J‐C Renauld. Wild type (WT, C57BL/6) mice were bred in‐house. All animal procedures were carried out in accordance with the European Council directives (86/609/EEC) and the Animal Scientific Procedures Act (UK) 1986 under the project license number 70/7800 to W.M.

#### Generation of IL‐1R1^fl/fl^ mice and conditional deletion of IL‐1R1

IL‐1R1 conditional (IL‐1R1^fl/fl^) mice were generated by gene targeting using BAC clones as the targeting vector from the C57BL/6J RPCI‐23 BAC library encoding two loxP sites flanked exon 5 of the *il1r1* gene (size of loxP‐flanked arm 1.2 kb), and subsequent homologous recombination in C57BL/6N embryonic stem (ES) cells. From correctly targeted C57BL/6N ES cells, as verified by southern blotting, chimeric mice were generated bred to C57BL/6 females. Germline transmission was identified by genotyping PCR sample analysis using a Caliper LabChip GX device (details are available upon request). Genotyping identification of IL‐1R1^fl/fl^ mice was carried out by PCR using the following primers; Forward: CTAGTCTGGTGGAACTTACATGC, depicted (1) in Fig. 1B; Reverse: AACTGAAAGCTCAGTTGTATACAGC, depicted (2) in Fig. 1B, on genomic DNA, in reaction mix composed of 5 μL PCR Buffer 10x (Invitrogen), 2 μL MgCl_2_ (50 mM), 1 μL dNTPs (10 mM), 1 μL of each primer (5 μM), 0,2 μL Taq (5 U/μL, Invitrogen), 37.8 μL H_2_O, 2 μL DNA, using the following amplification protocol (95°C 5 min; 95°C 30 s; 60°C 30 s; 72°C 1 min; 35 cycles; 72°C 10 min). Amplification product size obtained were as follow: wild type (267 bp), IL‐1R1^+/fl^ (267 bp + 432 bp), IL‐1R1^fl/fl^ (432 bp).

#### Generation of a new IL‐1R1^−/−^ mice

A new ubiquitous IL‐1R1^−/−^ mouse lacking all IL‐1 receptor isoforms (including IL‐1R1 and IL‐1R3) was generated by crossing IL‐1R1^fl/fl^ mice with mice expressing Cre recombinase under the control of the human keratin 14 promoter in oocytes as described 7, leading to genetic deletion in all tissues of exon 5 that encodes part of the extracellular region (Ig‐like‐C2 type 2) of IL‐1R1 and IL‐1R3. The deletion of exon 5 causes a frame shift from exon 4 to all downstream exons. Genotypic identification of exon 5 deletion in IL‐1R1^−/−^ mice was carried out by PCR on isolated genomic DNA using the following primers; Forward: CTAGTCTGGTGGAACTTACATGC, depicted (1) in Fig. 1B; Reverse: GATAAAGCAGAGCTGGAGACAGG, depicted (3) in Fig. 1B, in the same reaction mix as described above, and using the following amplification protocol (95°C 5 min; 95°C 30 s; 63°C 30 s; 72°C 1 min; 35 cycles; 72°C 10 min). In the IL‐1R1^−/−^ mice, one band showed at approximately 400 bp.

#### Generation of mice with hematopoetic system‐specific inactivation of IL‐1 receptor isoforms

Conditional deletion of IL‐1 receptor isoforms in hematopoetic cells was achieved by crossing IL‐1R1^fl/fl^ mice with mice expressing Cre recombinase under the control of the vav promoter 9, leading to deletion of all IL‐1 receptor isoforms in hematopoietic cells (IL‐1R1^fl/fl^ vav iCre+ mice). Genotypic identification of exon 5 deletion in IL‐1R1^fl/fl^ vav iCre+ mice was carried out as described for IL‐1R1^−/−^ mice. Genotypic identification of Cre recombinase allele was carried out by PCR using the following primers; Forward: AGATGCCAGGACATCAGGAACCTG; Reverse: ATCAGCCACACCAGACACAGAGATC using the following amplification protocol (94°C 5 min; 94°C 30 s; 56°C 30 s; 68°C 1 min; 35 cycles; 68°C 5 min). In IL‐1R1^fl/fl^ vav iCre+ mice, one band showed at 250 bp.

#### IL‐1R1 expression and cytokine expression in isolated spleen cells

Cells were isolated from the spleen of mice by homogenization through a 70 μm cell strainer (BD Biosciences) and centrifugation at 450 g for 5 min. Cell pellets were treated with 3 mL of erythrolysis lysing buffer (BD Biosciences) for 10 min at room temperature. The suspension was then washed and centrifuged at 450 *g* for 5 min. The cells were then resuspended in complete RPMI medium and counted. Spleen cells (5 × 10^6^ cells/mL) were plated in 96‐well culture plate and were stimulated with either 100 ng/mL LPS (sigma) or 20 ng/mL IL‐1β (R&D Systems) at 37°C for 24 h. Cell were collected and assayed for IL‐1R1 by Western blot analysis using a specific anti IL‐1R1 antibody (Abcam), and levels of inflammatory mediators in the culture supernatants were assayed by specific ELISAs for IL‐6 and MCP‐1 (BD Biosciences, R&D Systems).

#### Trichuris muris infection and measurement of Th1‐mediated immune response

Mice were infected by oral gavage with a low dose (approx. 20 or 30 infective *T. muris* eggs), as previously described 13. On day 21 postinfection, the mice were euthanized, and worm burden were counted in caecum and colon proximal to caecum as previously described 14. MLN cells were isolated by homogenization through a 70‐μm cell strainer (BD Biosciences) and centrifugation at 450 x *g* for 5 min. Cell were resuspended in complete RPMI medium and counted. MLN cells (5 × 10^6^ cells/mL) were plated in 96‐well culture plate and were restimulated for 4 h with 50 μg/mL parasite E/S antigen, after which culture supernatants were collected an assayed for IL‐17 and IL‐22 by ELISAs (BD Biosciences). *T. muris*‐specific antibody (IgG2a) levels were measured in the blood serum of infected mice using a specific ELISA. Nine fluorescence parameter flow cytometry was performed to isolate and count immune cells from the spleen, blood, and MLN using a LSR‐Fortessa FACS sorter and Cell Quest software (BD Biosciences). The gating strategy was as follows: dead cells were excluded based on Sideward scatter and Cy7 staining, and single cells were selected. A combination of two PE‐CY5‐conjugated antibodies specific for CD4 and CD8 was then used to distinguish between CD4 (medium) and CD8 (bright) cells. In the same window we used CD19 APC to stain and gate B cells (APC positive cells). Cells that were negative for CD4, CD8, and CD19 were gated as non‐B/non‐T cells, and were further distinguished into IgM only (most likely immature B cells) and IgM/IgD‐positive cells (most likely mature B cells) using PE‐conjugated IgM and pacific blue‐conjugated IgD. Non‐B/non‐T cells were further separated into monocytes (F4/40 positive, PE coupled), granulocytes (GR1 positive, PE coupled + pacific blue coupled), and NK cells (CD49 positive, pacific blue coupled).

## Statistical analyses

Statistical differences between three groups or more was determined using one‐way or two‐way ANOVA followed by a Tukey multiple comparison post hoc test. Significant difference between two groups was determined using Student's *t*‐test. Significant differences were described as *p < 0.05, **p < 0.01, ***p < 0.001.

## Conflict of interest

The authors declare no financial or commercial conflict of interest.

## Supporting information

As a service to our authors and readers, this journal provides supporting information supplied by the authors. Such materials are peer reviewed and may be re‐organized for online delivery, but are not copy‐edited or typeset. Technical support issues arising from supporting information (other than missing files) should be addressed to the authors.

Characterization of a conditional interleukin‐1 receptor 1 mouse mutant using the Cre/LoxP system

Specific gating strategy using nine fluorochrome labeling
The analysis of lymphocytes in the spleen (A), blood (B) and MLN (C)
B cells label gating strategy used for spleen (A) and BM (B) cells.
The analysis of B cells lymphocyte in the spleen and BM.
