# Supplementary material for: Characterization of a conditional interleukin‐1 receptor 1 mouse mutant using the Cre/LoxP system
Source: Eur J Immunol. 2016 Jan 18;46(4):912–8. doi: 10.1002/eji.201546075 (PMC4982085; doi:10.1002/eji.201546075)
Supplement: Supplementary file 2 — Specific gating strategy using nine fluorochrome labeling The analysis of lymphocytes in the spleen (A), blood (B) and MLN (C) B cells label gating strategy used for spleen (A) and BM (B) cells. The analysis of B cells lymphocyte in the spleen and BM. [file EJI-46-912-s002.pdf]

Supplementary Figure 1

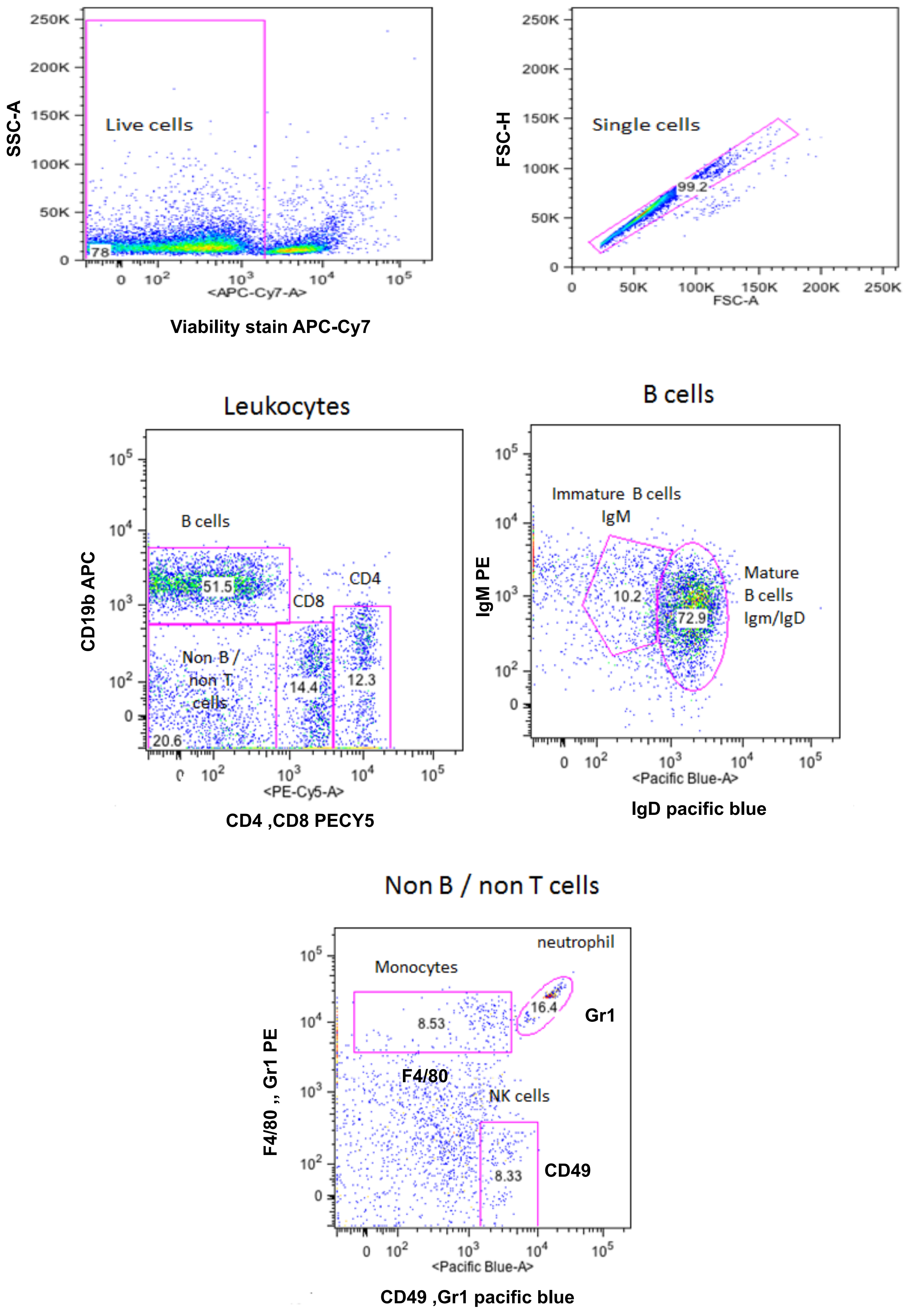

Supplementary Figure 2A

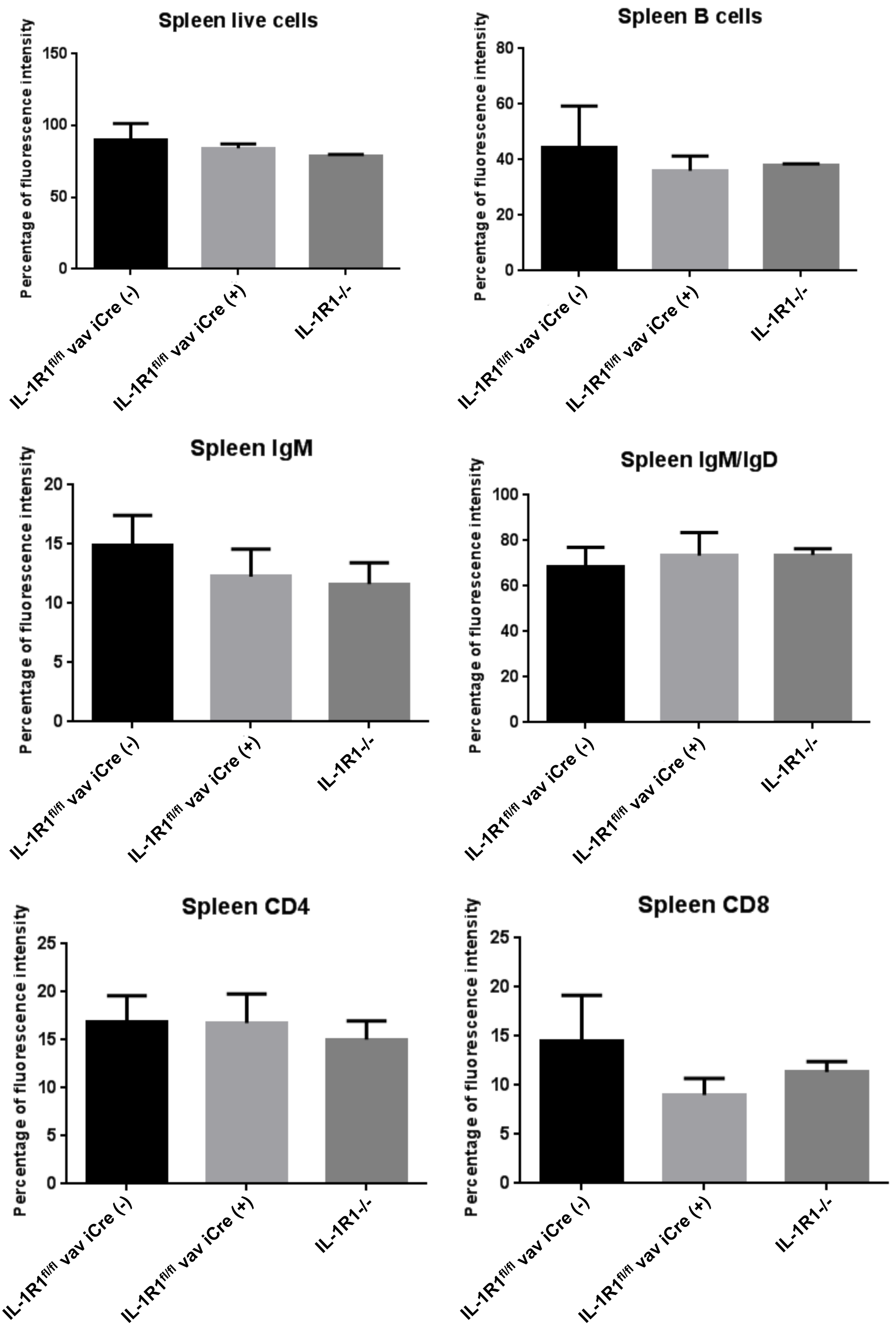

Supplementary Figure 2A (cont.)

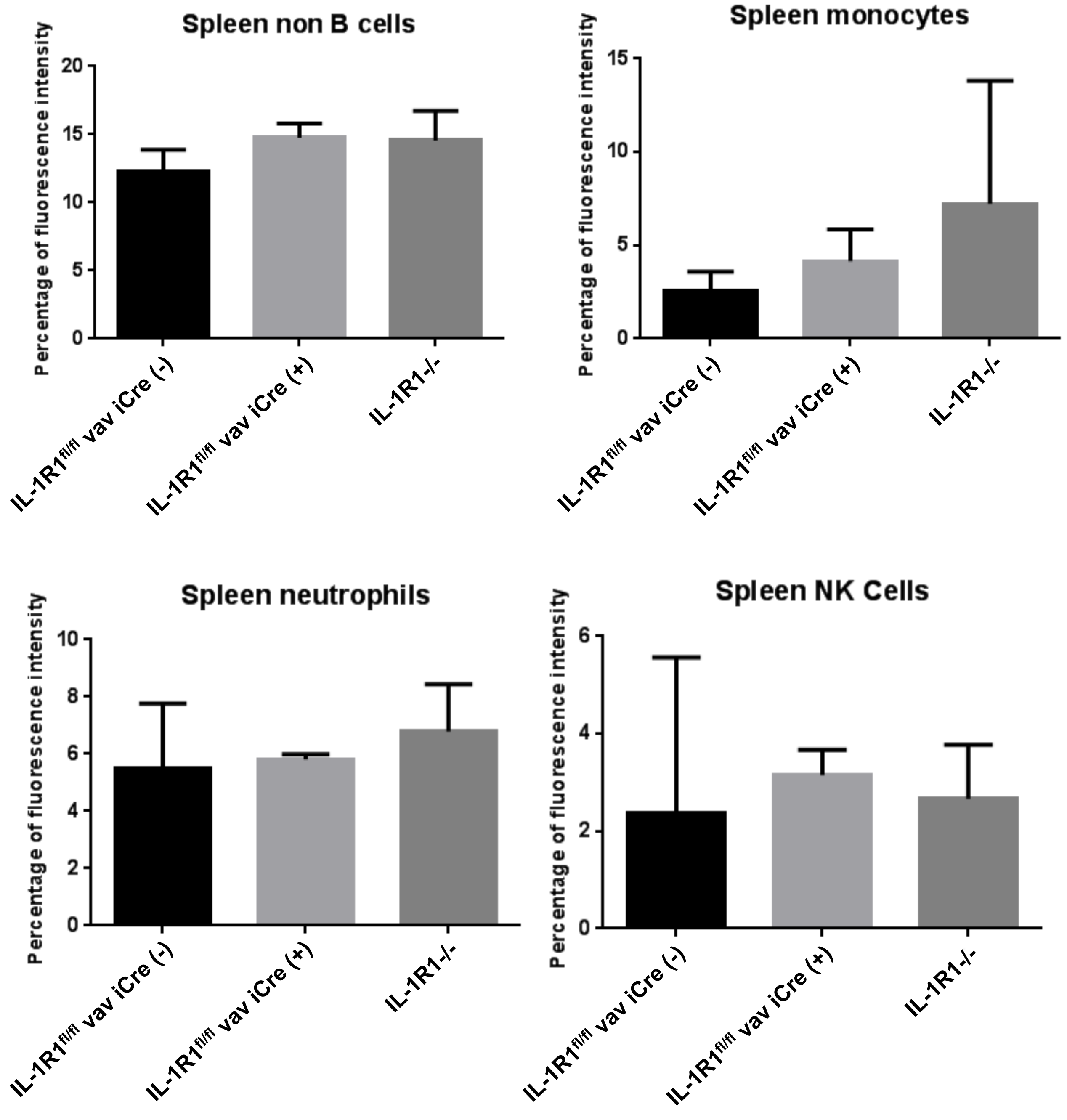

Supplementary Figure 2B

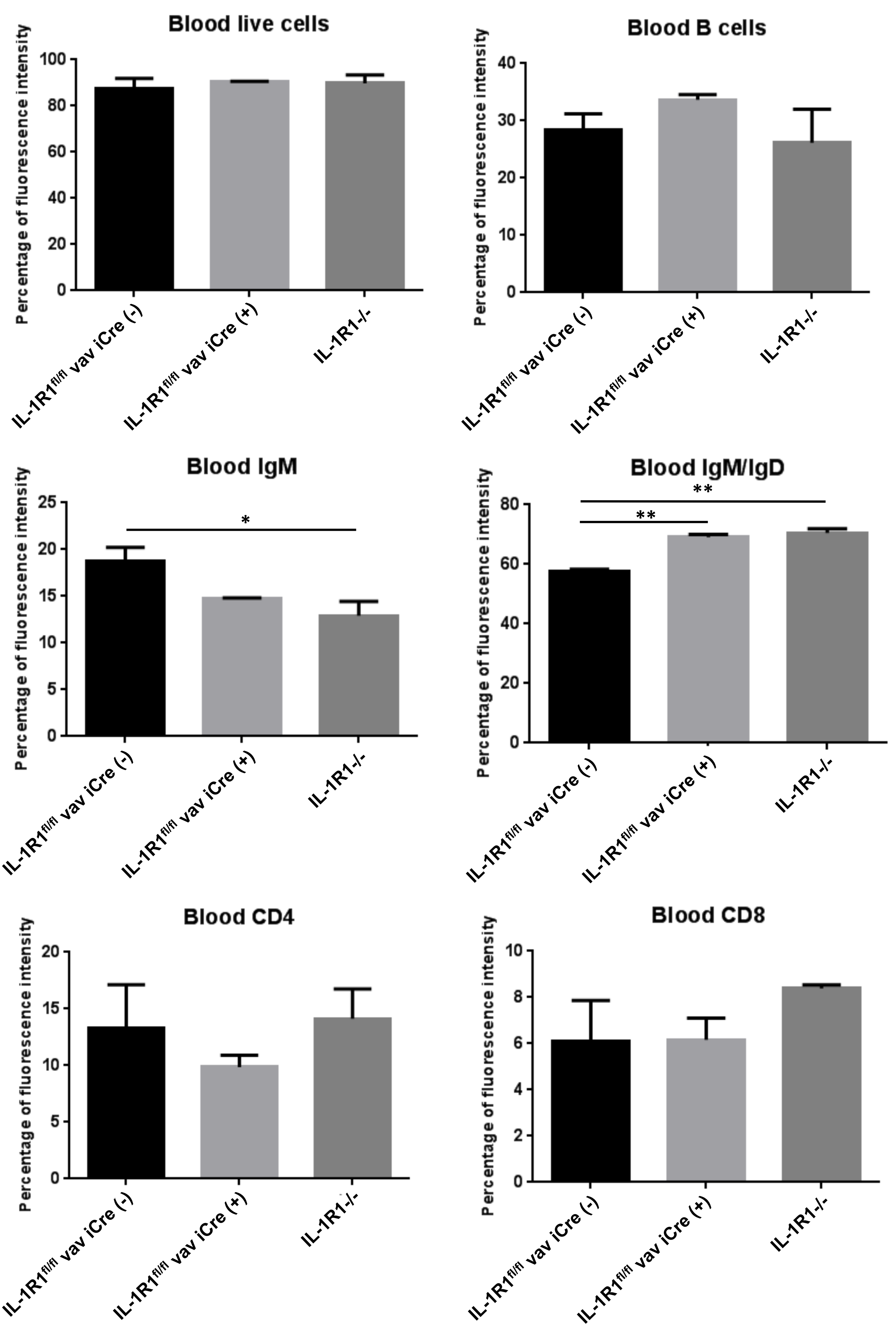

Supplementary Figure 2B (cont.)

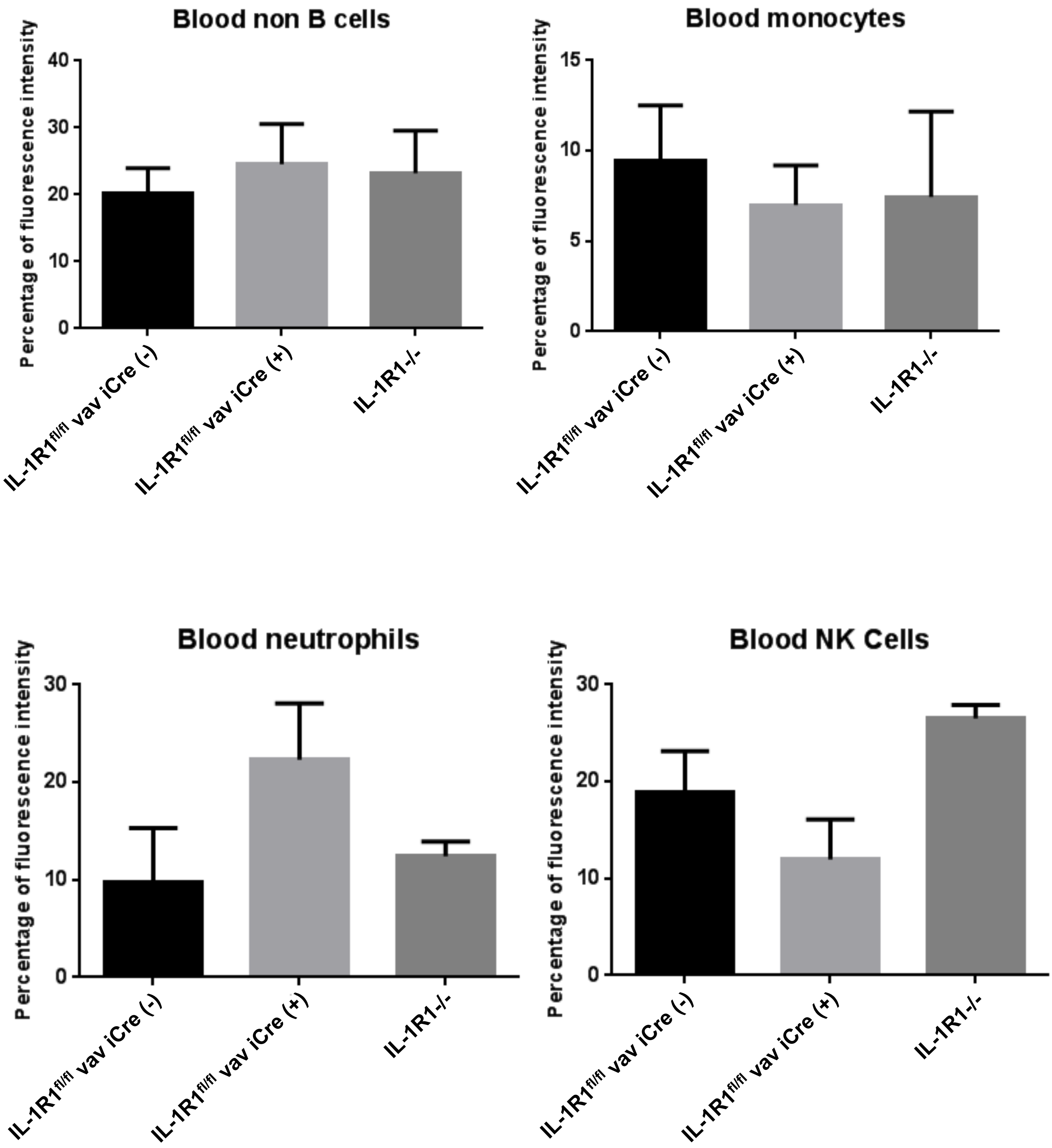

Supplementary Figure 2C

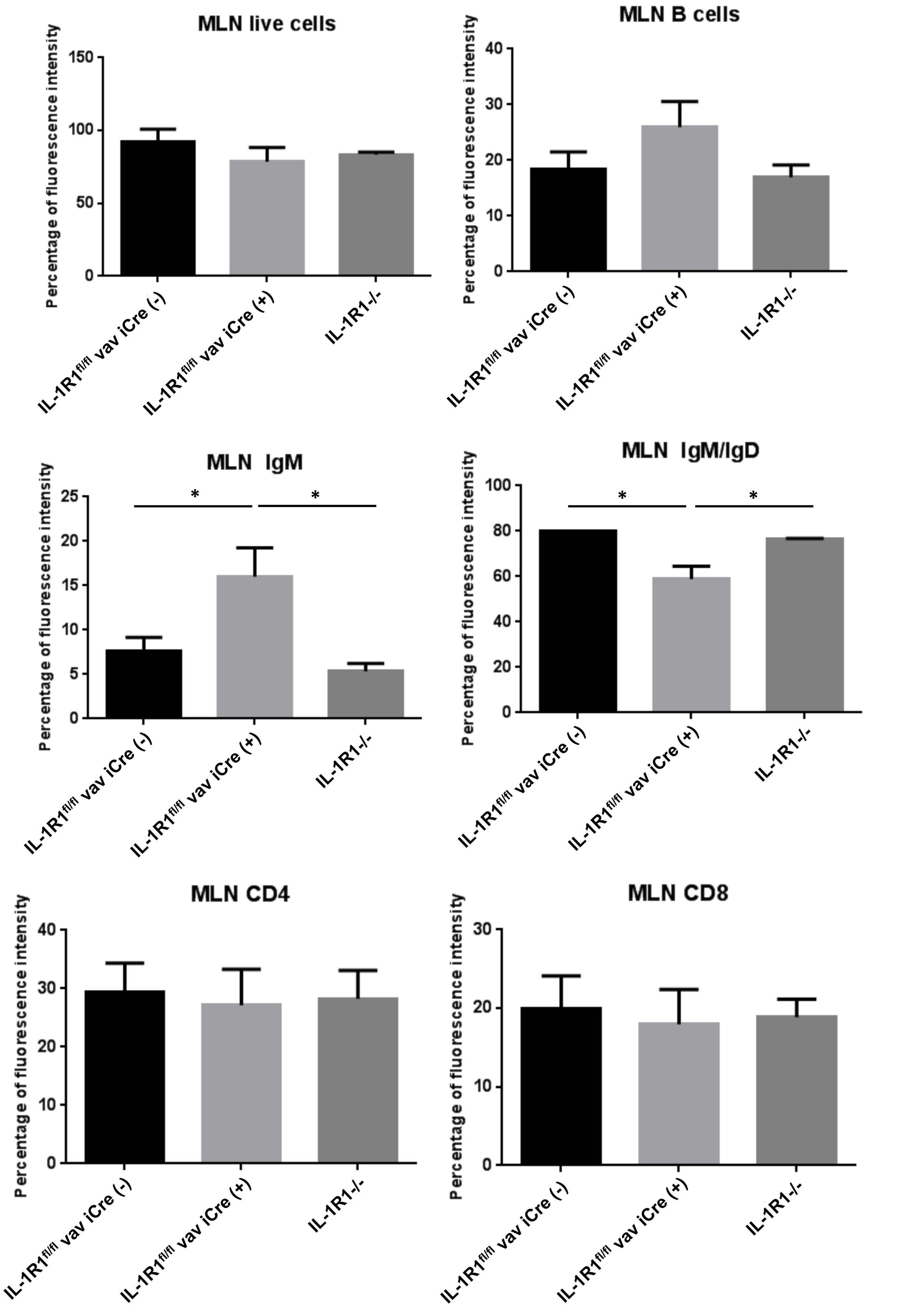

Supplementary Figure 2C (cont.)

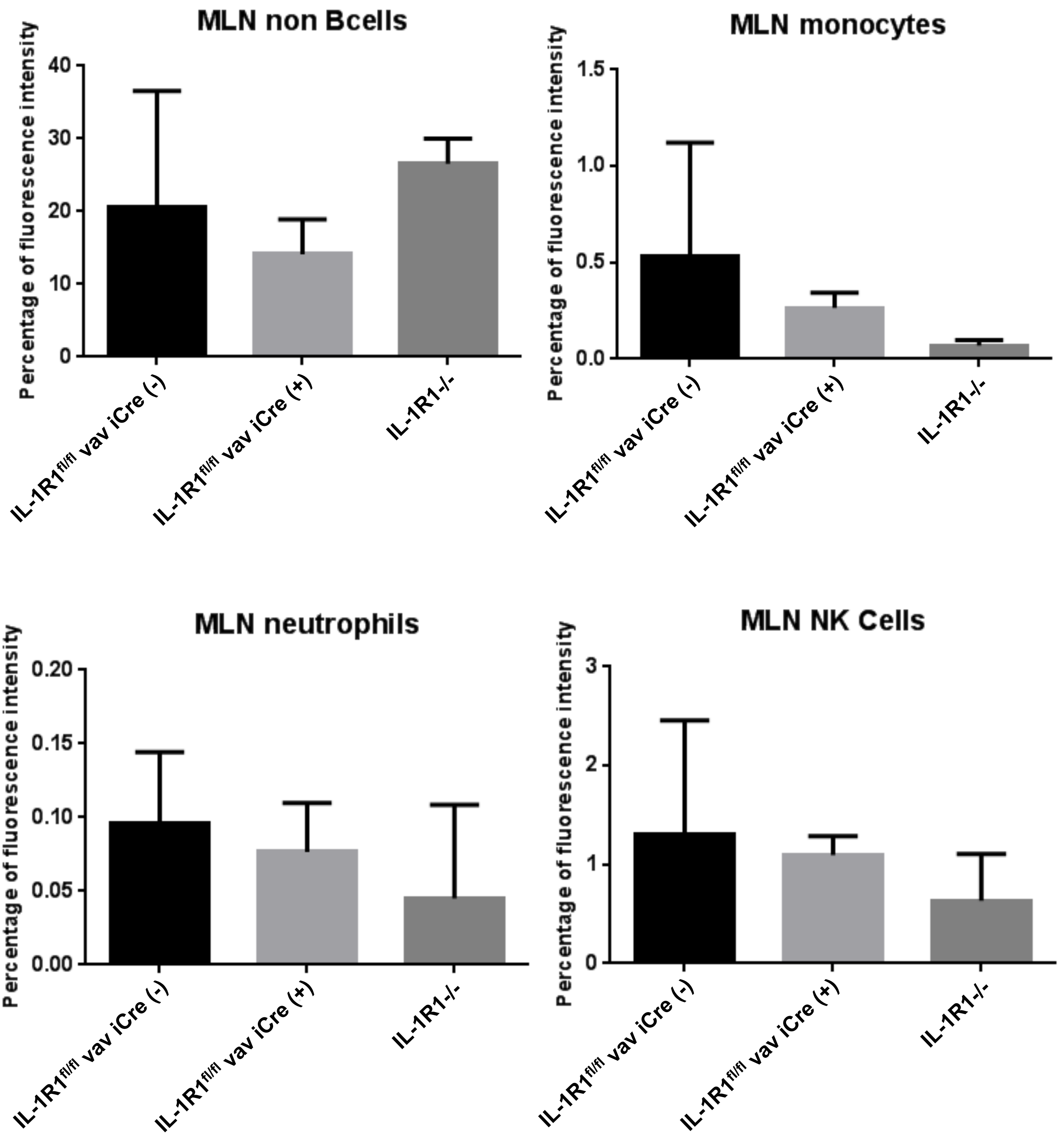

Supplementary Figure 3

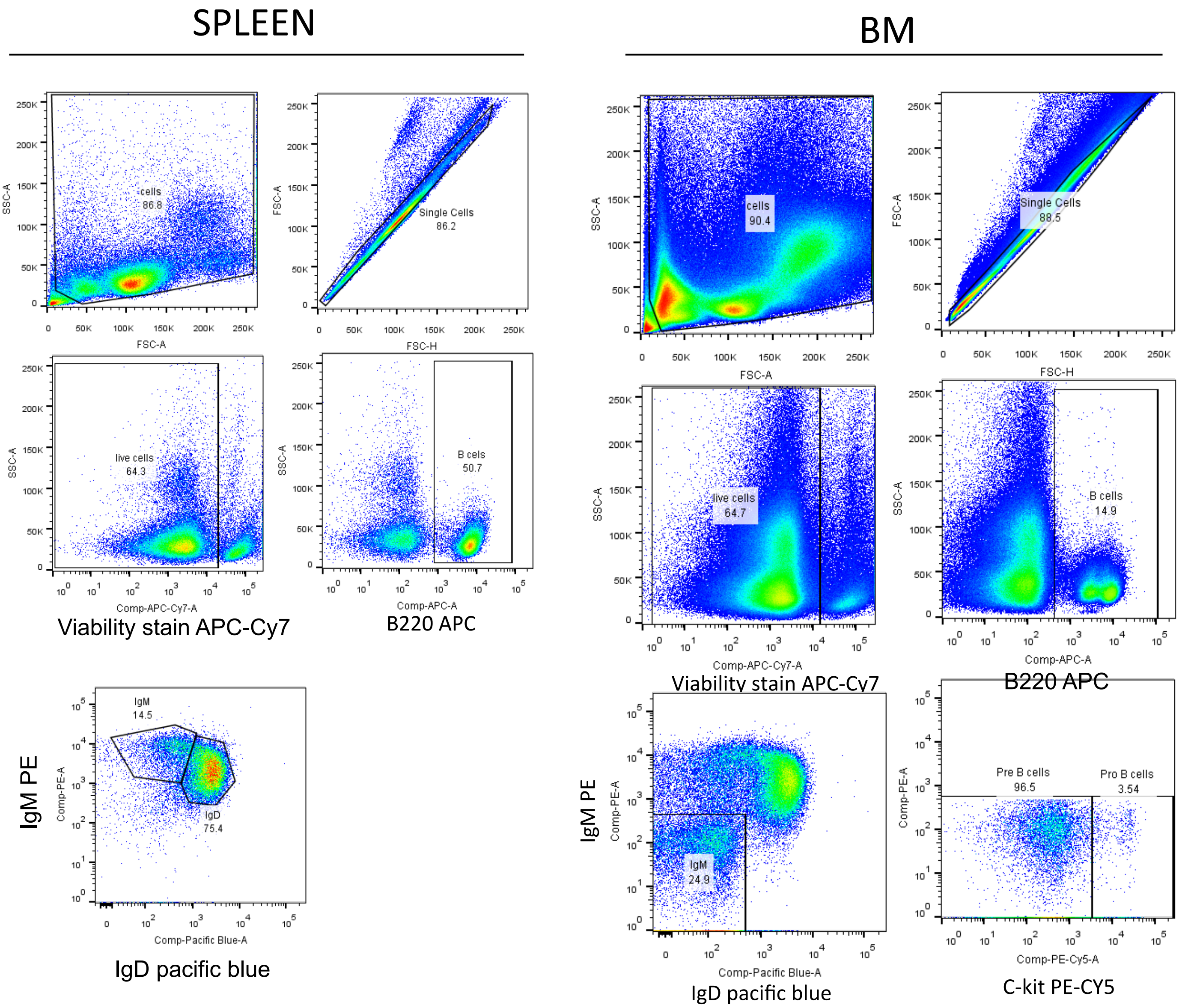

Supplementary Figure 4

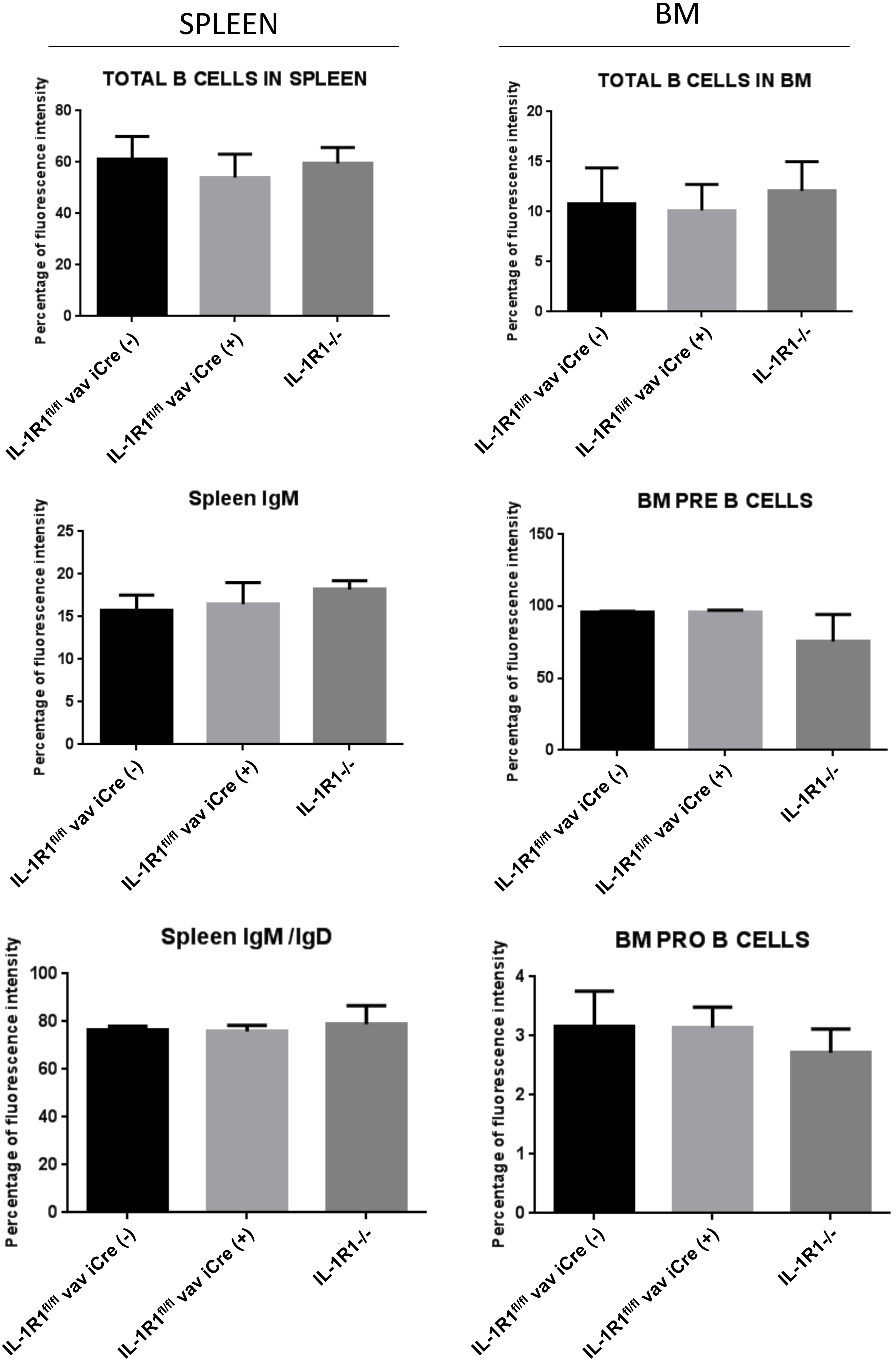

Supplementary Figure 1: Specific gating strategy using nine fluorochrome labeling. The spleen, blood and MLN cells were isolated from the IL-1R1$^{fl/fl}$ vav iCre-, IL-1R1$^{fl/fl}$ vav iCre+ and IL-1R1-/- mice. Leukocytes were separated and differentiated into T cells (CD4+ and CD8+) and were gated using PE-Cy5, B cells and non-B/non-T cells, and was gated using CD19-APC. Secondly, Cells were gated and differentiated into immature IgM using PE-IgM+, and mature B IgM+IgD+ cells by using (PE-IgM+ and Pacific Blue IgD+). Non-B/non-T cells were then separated into NK cells by using Pacific Blue CD49b+, macrophages/monocytes using PE-F4/80+, and into neutrophils by using Pacific Blue Gr1+ PE-Gr-1+. Cells were acquired on a LSR-Fortessa machine (BD Biosciences) and the data was analysed using FlowJo.

Supplementary Figure 2: The analysis of lymphocytes in the spleen (A), blood (B) and MLN (C). Cells were isolated from IL-1R1$^{fl/fl}$ vav iCre-, IL-1R1$^{fl/fl}$ vav iCre + and IL-1R1-/- mice and stained using the nine stain protocol. Cells were acquired on the LSR-Fortessa machine (BD Biosciences) and the data was analysed using FlowJo. The data are presented as mean percentages (+/- SEM), n=3. To analyse the data, a one-way ANOVA for each lymphocyte was conducted, followed by a Tukey's multiple comparison post hoc test. (*P<0.05, **P<0.01).

Supplementary Figure 3: B cells label gating strategy used for spleen (A) and BM (B) cells. Cells were isolated from IL-1R1$^{fl/fl}$ vav iCre-, IL-1R1$^{fl/fl}$ vav iCre + and IL-1R1-/- mice. In both the spleen and BM cells, the B cells were identified and gated using APC-B220+. Subsequently, B cells were gated and differentiated into immature B cells IgM+IgD- by using PE-IgM+ and mature B cells IgM+IgD+ cells using PE-IgM+ and pacific blue IgD+. The IgM+ cells were separated and differentiated only in BM into pre-B cells (C-kit-) and pro -B cells (C-kit+) using C-kit PE-CY5. Cells were acquired on a LSR-Fortessa machine (BD Biosciences) and the data was analysed using FlowJo.

Supplementary Figure 4: The analysis of B cells lymphocyte in the spleen and BM. Spleen (A) and BM (B) cells were isolated from IL-1R1$^{fl/fl}$ vav iCre-, IL-1R1$^{fl/fl}$ vav iCre + and IL-1R1-/- mice and stained using the B development stain protocol. Cells were acquired on the LSR-Fortessa machine (BD Biosciences) and the data was analysed using FlowJo. The data are presented as mean percentages (+/- SEM), n=3. To analyse the data, a one-way ANOVA for each lymphocyte was conducted, followed by a Tukey's multiple comparison post hoc test.
