# Supplementary material for: Characterization of a conditional interleukin‐1 receptor 1 mouse mutant using the Cre/LoxP system
Source: Eur J Immunol. 2016 Jan 18;46(4):912–8. doi: 10.1002/eji.201546075 (PMC4982085; doi:10.1002/eji.201546075)
Supplement: Supplementary file 1 — Characterization of a conditional interleukin‐1 receptor 1 mouse mutant using the Cre/LoxP system [file EJI-46-912-s001.pdf]

European Journal of Immunology

Peer review correspondence

Characterization of a conditional interleukin-1 receptor 1 mouse mutant using the Cre/LoxP system

Wesam H. Abdulaal, Catherine R. Walker, Ryan Costello, Elena Redondo-Castro, Ilgiz A. Mufazalov, Athina Papaemmanouil, Nancy J. Rothwell, Stuart M. Allan, Ari Waisman, Emmanuel Pinteaux and Werner Müller

Corresponding author: Emmanuel Pinteaux, Faculty of Life Sciences, University of Manchester, AV Hill Building, Oxford Road, Manchester, United Kingdom

---

Review Timeline:	Submission date:	16 September 2015
	First editorial decision:	12 October 2015
	First revision received:	20 October 2015
	Second editorial decision:	12 November 2015
	Second revision received:	22 November 2015
	Accepted:	15 December 2015

Handling Executive Committee members: Prof. Andreas Radbruch

Please note that the correspondence below does not include the standard editorial instructions regarding preparation and submission of revised manuscripts, only the scientific revisions requested and addressed.

First Editorial Decision – 12 October 2015

Dear Dr. Pinteaux,

Manuscript ID eji.201546075 entitled "Characterization of a conditional interleukin-1 receptor 1 mouse mutant using the Cre/LoxP system" which you submitted to the European Journal of Immunology has been reviewed. The comments of the referees are included at the bottom of this letter.

You will see that referee 1 has some deep concerns regarding the lack of biological relevance of the mouse presented, and feels that the data presented in the disease model are weak. This referee recommended that we reject the manuscript; however we and the Executive Editor feel that, given this manuscript was submitted under scoop protection, the recommendation is harsh. However, given that some of this referee's concerns are echoed by referee 2, we would consider a revised version of your

manuscript that addresses the concerns of both referees, as far as possible in terms of clarifying the construction, expression and basic biology of your conditional knockout. We ask for no new data on the disease model, but do ask that you tone down some statements, check and correct others and discuss the limitations of what you have measured in the present work.

Should you disagree with any of the referees' concerns, you should address this in your point-by-point response and provide solid scientific reasons for why you will not make the requested changes.

You should also pay close attention to the editorial comments included below. *In particular, please edit your figure legends to follow Journal standards as outlined in the editorial comments. Failure to do this will result in delays in the re-review process.*

Please note that submitting a revision of your manuscript does not guarantee eventual acceptance, and that your revision will be re-reviewed by the referees before a decision is rendered.

If the revision of the paper is expected to take more than three months, please inform the editorial office. Revisions taking longer than six months may be assessed by new referees to ensure the relevance and timeliness of the data.

Once again, thank you for submitting your manuscript to European Journal of Immunology and we look forward to receiving your revision.

Yours sincerely,
Karen Chu

On behalf of Prof. Andreas Radbruch

Dr. Karen Chu
Editorial Office
European Journal of Immunology
e-mail: ejied@wiley.com
www.eji-journal.eu

****

Reviewer: 1

Comments to the Author

European Journal of Immunology

The authors report the characteristics of a novel floxed IL-1R1 mouse line and some preliminary results in constitutive IL-1R1 KO mice and in mice in which IL-1R1 has been specifically targeted in hematopoietic cells. This new floxed mouse line will certainly provide an important tool to examine the cell-specific effect of IL-1 in different inflammatory models.

However I have several concerns regarding the presentation of the data. Furthermore, most of the data are focused on the characterization of the mice and only a minor section is devoted to the biological relevance in a disease model. In addition, the results related to the disease model are weak and include many mistakes.

Major concerns:

Cre recombinase expressed under the control of the human K14 promoter is usually used to target the expression of genes in keratinocytes and various epithelial cells. Here the authors have used a human K14 promoter-Cre transgenic mouse line to obtain constitutive IL-1R1 KO mice. The authors should provide more explanation to the readers on this mouse line and the difference with other reports on human K14 promoter-Cre transgenic mice.

The authors should clearly show the primers used for genotyping floxed and KO mice within the figure representing the gene (Fig. 1).

The PCR results shown in Fig. 1C should more precise. In some cases the authors used sometimes the term +/+ and sometimes WT. For clarity, they should use the same terminology. Similarly, IL-1R1fl/fl vav iCre- should be rather described as IL-1R1fl/fl. The presentation of the PCR should be consistent in the different panels. The referee does not understand why in some cases bands of +/+ and IL-1R1fl/fl are different (left panel), whereas in the middle panel they look similar. Similarly, it is better to run the PCR results for IL-1R1fl/fl x K14 Cre in the same gel as IL-1R1fl/fl vav iCre since the band should have a similar size.

In addition, the referee would like to see some controls from different cells/tissues showing that the deletion is selective or in contrast, ubiquitous.

The results of the WB showing that IL-1R1 is present in LPS-stimulated cells but not in unstimulated or IL-1-stimulated cells is quite strange. This result suggests that LPS stimulates the expression of IL-1R1 in some cell types. Is this notion supported by other data? The authors must use other approaches to confirm this finding and determine which cells up-regulate the expression of IL-1R1 in response to LPS?

The authors have used a model of parasitic infection to examine the role of IL-1 responses in hematopoietic cells using IL-1R1fl/fl vav iCre mice. They mention that the “lack of IL-1R1 in hematopoietic system leads to a stronger Th1 response” but there is no evidence for this statement.

They also mention that the IgG2a response is stronger in KO mice whereas they show opposite results (Fig 3B). Finally, they refer to Th1 response by showing IL-17 and IL-22 productions, which is completely wrong.

The last paragraph of the Results and Discussion section in completely speculative when they mention that IL-17 is derived from T cells and IL-22 from other cells. The authors should provide some evidence. Since IL-22 seems to be critical in this model it is important to better characterize this point.

The section “concluding remarks” is too vague and pointless. Indeed, the authors mention some of the other lines that they have been able to generate without any connection to the findings included in the manuscript.

Minor concern:
Page 5, paragraph 1: the authors mention: “the production of any of the three known transcribed forms of the il-1r1 gene”. To which three transcripts are the referring to? I think that there are only two transcripts: il1r1 and il1r3.
In addition, il-1r1 should rather read Il1r1

Reviewer: 2

Comments to the Author
Abdulaal and colleagues report the generation of IL-1R1 fl/fl animals in which exon 5 of the il-1r1 gene is flanked by LoxP sites. In contrast to previously published IL-1R1 germline knockout mice the new mouse strain might offer the possibility to interrupt expression of all known IL-1R1 variants in a cell type specific manner. Nematode infection of mice in which IL-1R1 expression is ablated in hematopoietic cells showed that IL-1 receptor signaling is essential for the proper control of the parasite.

As IL-1 receptor signaling is essential in many inflammatory disorders, the new mouse strain is of broad interest. However, the current study falls short in several aspects that should be further addressed:

Major Comments:
- loxP sites in the il-1r1 introns flanking exon 5 and “subtle sequence modifications in both exon 5 and exon 6” (page 5; line 18f) were introduced by the authors. To exclude functional impairment of the il-1r1 gene due to the sequence mutations wt animals should be included in all analysis in order to demonstrate that IL-1R1 fl/fl animals (without Cre expression) are comparable to wt animals in the functional studies.
- Deletion of il-1r1 exon 5 should not result in production of the 3 previously described IL-1R1 gene transcripts, but experimental evidence is not provided.

European Journal of Immunology

- The contamination of splenocytes isolated from IL-1R1 fl/fl vav iCre (+) animals with non-hematopoietic cells precludes the interpretation of data obtained after LPS stimulation. The analysis of enriched cell populations (based on e.g. magnetic bead purification or FACS sorting) will allow the authors to exclude the presence of “escapees” in the hematopoietic system.
- In contrast to the authors’ statement that IL-1β induces IL-1 receptor expression on many cell types (page 5, line 48f) it does not induce receptor expression on the analyzed splenocytes (Figure 1D). The authors might want to comment on the result and why they included this “positive control” in the manuscript.
- The selection of TNFα, IL-6 and MCP-1 (Figure 2) as readout for IL-1R1 activation after LPS and IL-1β stimulation needs an explanation. Furthermore, TNFα release does not seem to be significantly increased after IL-1β treatment in IL-1R1 fl/fl vav iCre (-) control animals and thus the results are difficult to interpret.
- The authors might want to move the LPS data from Figure 2 in the supplement as the effects of LPS stimulation on IL-1R1 deficient hematopoietic cells are masked by the presence of IL-1R1 competent cells.
- IL-22 expression is significantly different between wt and IL-1R1 fl/fl vav iCre (-) animals (Figure 3). Does this reflect the effect of the targeted IL-1R1 allele?

Minor Comments:
- The authors should avoid to use the same abbreviation (= IL-1R1 -/-) for the previously described KO animals and the newly generated IL-1R1 fl/fl Keratin 14-Cre mice.
- The number of worms in the caecum/proximal colon varies significantly between control animals in the different experiments (Figure 3A). Do the authors have any explanation?
- Page 5, line 22f: the authors might rewrite the sentence “ By positioning the loxP sites….”
- Page 5, line 54: which genotype corresponds to “IL-1R1 fl/fl vav iCre+ homozygous mice”?
- Suppl. Figure 3: immature B cells might not be the major population in splenic IgM+, IgD- B cells (see figure legend).
- Suppl. Figure 5 “cells” instead of “celss”.

<u>First revision – authors’ response – 20 October 2015</u>

Reviewer: 1

The authors report the characteristics of a novel floxed IL-1R1 mouse line and some preliminary results in constitutive IL-1R1 KO mice and in mice in which IL-1R1 has been specifically targeted in hematopoietic cells. This new floxed mouse line will certainly provide an important tool to examine the cell-specific effect of IL-1 in different inflammatory models. However I have several concerns regarding the presentation of the data. Furthermore, most of the data are focused on the characterization of the mice and only a minor

European Journal of Immunology

section is devoted to the biological relevance in a disease model. In addition, the results related to the disease model are weak and include many mistakes.

Major concerns:

- Cre recombinase expressed under the control of the human K14 promoter is usually used to target the expression of genes in keratinocytes and various epithelial cells. Here the authors have used a human K14 promoter-Cre transgenic mouse line to obtain constitutive IL-1R1 KO mice. The authors should provide more explanation to the readers on this mouse line and the difference with other reports on human K14 promoter-Cre transgenic mice.

Answer: We generated a series of K14 promotor driven cre transgenic mouse lines. The lines were published in Hafner M, Wenk J, Nenci A, Pasparakis M, Scharffetter-Kochanek K, Smyth N, Peters T, Kess D, Holtkötter O, Shephard P, Kudlow JE, Smola H, Haase I, Schippers A, Krieg T, Müller W. Keratin 14 Cre transgenic mice authenticate keratin 14 as an oocyte-expressed protein. Genesis. 2004 Apr;38(4):176-81, reference 7 in the manuscript. When we generated such lines we tested the gene deletion efficiencies of the K14 cre transgene using a loxP flanked allele. We noticed that when we used male cre transgenic mice, the gene deletion in the offspring is restricted to the keratinocytes as expected. However, when female cre transgenic mice were used, we noticed a very efficient germline deletion of LoxP flanked transgenes. The deletion of the loxP flanked region happened independent of the inheritance of the K14 cre transgene, suggesting that the cre protein is already preformed in the oocytes before fertilisation. We subsequently showed that also the K14 protein is expressed in oocytes suggesting that we were observing a normal function of the K14 promoter. We assume that also other K14 cre lines will show similar properties, may do so to a different extent.

- The authors should clearly show the primers used for genotyping floxed and KO mice within the figure representing the gene (Fig. 1).

Answer: The primers were already depicted in the Figure 1B, as semi-arrows. We have now labelled each primer, as (1), (2) and (3) in the figure. We have now modified the section “Genotyping protocols” in the Supplementary Methods so that each labelled primer is provided with each corresponding sequence. The figure legend has also been modified accordingly.

- The PCR results shown in Fig. 1C should more precise. In some cases the authors used sometimes the term +/+ and sometimes WT. For clarity, they should use the same terminology. Similarly, IL-1R1fl/fl vav iCre- should be rather described as IL-1R1fl/fl. The presentation of the PCR should be consistent in the different panels. The referee does not understand why in some cases bands of +/+ and IL-1R1fl/fl are different (left panel), whereas in the middle panel they look similar. Similarly, it is better to run the PCR results for IL-1R1fl/fl x K14 Cre in the same gel as IL-1R1fl/fl vav iCre since the band should have a similar size.

European Journal of Immunology

Answer: We agree with the reviewer that the use of +/+ and WT is inconsistent. To this end, we have changed “WT” to “+/+” in the middle panel of figure 1C, and now believe that the representation of the PCR is consistent in the different panels. Regarding the second point, bands of +/+ and IL-1R1fl/fl are different in the left panel because the PCR genotyping protocol used in the left panel is to identify insertion of LoxP sites on each side of exon 5. Here, the PCR results shows a band of bigger size due to LoxP site insertion compared to a wild-type (+/+) where no insertion occurs. In the middle panel, bands seen in lanes 1 and 2 are artefact of amplification, but what is warrant of exon 5 deletion and full recombination is the appearance of a strong band in IL-1R1fl/fl vav iCre + mice due to successful recombination which allows Forward and Reverse primers ((1) and (3)) to work when exon 5 is deleted. Regarding the third point, PCR results could have been run in the same gel, but the generation of the two mice where achieved at different time and separate genotyping PCR where carried out. This does not impair the validity of the results since appropriate controls were always included.

- In addition, the referee would like to see some controls from different cells/tissues showing that the deletion is selective or in contrast, ubiquitous.
Answer: We have unpublished data showing that in the new IL-1R1-/- the deletion is ubiquitous, and are currently validating the selective specificity of exon 5 deletion using another Cre mice (endothelial specific Cre mice as well as CD4 and CD14 Cre mice). We wish to keep those data that will be published in the corresponding papers.

- The results of the WB showing that IL-1R1 is present in LPS-stimulated cells but not in unstimulated or IL-1-stimulated cells is quite strange. This result suggests that LPS stimulates the expression of IL-1R1 in some cell types. Is this notion supported by other data? The authors must use other approaches to confirm this finding and determine which cells up-regulate the expression of IL-1R1 in response to LPS?
Answer: We are aware to the presence of the residual band detected by the IL-1R1 specific antibody, when spleen cells of the IL-1R1 fl/fl x vav iCre+ mice are stimulated with LPS. We do not see a band, when spleen cells from the complete IL1-1R1 deficient mouse are used. We could have better purified cells from the spleen and repeated the experiment. At the time, we were confident that the IL-1R1 gene is deleted in the IL-1R1 fl/fl x vav iCre+ mice, also because we were able to clone the IL-1R1 delta allele and verify it by sequence analysis.

- The authors have used a model of parasitic infection to examine the role of IL-1 responses in hematopoietic cells using IL-1R1fl/fl vav iCre mice. They mention that the “lack of IL-1R1 in hematopoietic system leads to a stronger Th1 response” but there is no evidence for this statement.
They also mention that the IgG2a response is stronger in KO mice whereas they show opposite results (Fig 3B). Finally, they refer to Th1 response by showing IL-17 and IL-22 productions, which is completely wrong.

Answer: We agree with the reviewer that the wording of our data is confusing and contradictory. Just to clarify; the nature of the immune response to T. muris is reflected in the antibody class of the parasite specific antibody produced during the infection. A high level of IgG1 antibody and low level of IgG2a antibodies indicates a Th2 dominated response and high level of IgG2a antibodies versus low level of IgG1 antibodies is indicative of a Th1 dominated response. Also worms are expelled in case of a Th2 dominated response but remain in the animal in case of a Th1 dominated response. We have therefore reworded the description of our data to clearly explain that in response to T. muris infection, a strong Th1 response is mounted, and that this response is reduced in the IL-1R1fl/fl vav iCre+ and IL-1R1-/- mice. Increased worm burden in the the IL-1R1fl/fl vav iCre+ is explained by the reduced IL-22 levels in the IL-1R1fl/fl vav iCre+, as previously reported (Sovran, B., et al., IL-22-STAT3 Pathway Plays a Key Role in the Maintenance of Ileal Homeostasis in Mice Lacking Secreted Mucus Barrier. Inflamm Bowel Dis, 2015. 21(3): p. 531-42.), and data are provided in Figure 3C. As a result of those changes, the figure numbering in figure 3 has been modified and the figure legend has been amended accordingly.

- The last paragraph of the Results and Discussion section in completely speculative when they mention that IL-17 is derived from T cells and IL-22 from other cells. The authors should provide some evidence. Since IL-22 seems to be critical in this model it is important to better characterize this point.
Answer: We agree with the reviewer that the statement provided is very speculative. We have now provided data using mice in which IL-1R1 is deleted in T cells (by crossing IL-1R1fl/fl mice with CD4cre mice and show that IL-17 is indeed derived from T cells, whereas IL-22 is not. We have inserted these data in the text between brackets.

- The section “concluding remarks” is too vague and pointless. Indeed, the authors mention some of the other lines that they have been able to generate without any connection to the findings included in the manuscript.
Answer: We have modify the concluding remarks of the manuscript to focus more on the content of the manuscript and not on mention other projects using the conditional cytokine receptor mouse mutants.

Minor concern:
- Page 5, paragraph 1: the authors mention: “the production of any of the three known transcribed forms of the il-1r1 gene”. To which three transcripts are the referring to? I think that there are only two transcripts: il1r1 and il1r3.
Answer: The Il1r1 gene is depicted in the NCBI database with the ID 16177. The current entry from October 2015 indicates in fact four different transcripts one of which was found only one time so far. The other three transcripts, referred to in the manuscripts have been found more than one time (http://www.ncbi.nlm.nih.gov/gene/16177). In our study, we have targeted the two known “functional” transcripts. We have clarified this point by inserting the word "functional" to say “the two previously described functional IL-1R1 gene transcripts”.

- In addition, il-1r1 should rather read Il1r1
Answer: We have now corrected this in the manuscript accordingly.

Reviewer: 2

Abdulaal and colleagues report the generation of IL-1R1 fl/fl animals in which exon 5 of the il-1r1 gene is flanked by LoxP sites. In contrast to previously published IL-1R1 germline knockout mice the new mouse strain might offer the possibility to interrupt expression of all known IL-1R1 variants in a cell type specific manner. Nematode infection of mice in which IL-1R1 expression is ablated in hematopoietic cells showed that IL-1 receptor signaling is essential for the proper control of the parasite.

As IL-1 receptor signaling is essential in many inflammatory disorders, the new mouse strain is of broad interest. However, the current study falls short in several aspects that should be further addressed:

Major Comments:
- loxP sites in the il-1r1 introns flanking exon 5 and “subtle sequence modifications in both exon 5 and exon 6” (page 5; line 18f) were introduced by the authors. To exclude functional impairment of the il-1r1 gene due to the sequence mutations wt animals should be included in all analysis in order to demonstrate that IL-1R1 fl/fl animals (without Cre expression) are comparable to wt animals in the functional studies.
Answer: We understand that one should carefully compare the fl/fl mutants to controls. Within the limitations of the current experiments we have not observed a major phenotype in the IL-1R1fl/fl mutant compared to controls. We have additional data to show that IL-1 responses are identical between wild type and IL-1R1fl/fl brain cells, but because those data do not fall within the remit of the current paper, they cannot be included here. Instead we provide a short inserting in the text reflecting the concern.

- Deletion of il-1r1 exon 5 should not result in production of the 3 previously described IL-1R1 gene transcripts, but experimental evidence is not provided.
Answer: It is very difficult to provide experimental evidence for the lack of all three transcripts. What we have done was to clone the modified allele, sequenced it and observed the expected frameshift modification. We have clarified the point we would like to make by inserting the word "functional" to say “the two previously described functional IL-1R1 gene transcripts.

- The contamination of splenocytes isolated from IL-1R1 fl/fl vav iCre (+) animals with non-hematopoietic cells precludes the interpretation of data obtained after LPS stimulation. The analysis of enriched cell populations (based on e.g. magnetic bead purification or FACS sorting) will allow the authors to exclude the presence of “escapees” in the hematopoietic system.
Answer: We agree with this point, and this has been addressed in our response to reviewer 1.

European Journal of Immunology

- In contrast to the authors' statement that IL-1β induces IL-1 receptor expression on many cell types (page 5, line 48f) it does not induce receptor expression on the analyzed splenocytes (Figure 1D). The authors might want to comment on the result and why they included this "positive control" in the manuscript.
Answer: Since IL-1β and LPS are known inducers of IL-1R1 expression, we included these controls to ensure that the il1r1 gene was fully inactivated (i.e. no residual functional gene after exon 5 deletion). We have slightly reworded the text.

- The selection of TNFα, IL-6 and MCP-1 (Figure 2) as readout for IL-1R1 activation after LPS and IL-1β stimulation needs an explanation. Furthermore, TNFα release does not seem to be significantly increased after IL-1β treatment in IL-1R1 fl/fl vav iCre (-) control animals and thus the results are difficult to interpret.
Answer: TNFα, IL-6 and MCP-1 are very well known downstream inflammatory mediators expressed in response to IL-1β and in many cell types. We could have chosen other inflammatory markers of IL-1R1 activation and we anticipate that similar responses will be observed.
Yes, this is true that TNFa release is not significantly increased after IL-1β, but although non-significant, there is a trend to increase. Maybe this is masked by high constitutive expression, but we show that this expression is completely reduced in the IL-1R1 fl/fl vav iCre (+) mice.

- The authors might want to move the LPS data from Figure 2 in the supplement as the effects of LPS stimulation on IL-1R1 deficient hematopoietic cells are masked by the presence of IL-1R1 competent cells.
Answer: We believe that the LPS data are important because they show that if exon 5 is deleted, the receptor won't be expressed but the IL-1R1-dependent downstream signalling pathways are intact since cells still respond to LPS (given that TLR4 uses same signalling mechanisms to that of IL-1R1).

- IL-22 expression is significantly different between wt and IL-1R1 fl/fl vav iCre (-) animals (Figure 3). Does this reflect the effect of the targeted IL-1R1 allele?
Answer: The reviewer is making an incorrect statement, because we are not making a statistical comparison between wt and IL-1R1 fl/fl vav iCre (-) A direct comparison between IL-1R1 fl/fl vav iCre (-) animals and wild type mice cannot be made because the wild type used was the wild type corresponding to IL-22-/- and not IL-1R1 fl/fl vav iCre (-).

Minor Comments:
- The authors should avoid to use the same abbreviation (= IL-1R1 -/-) for the previously described KO animals and the newly generated IL-1R1 fl/fl Keratin 14-Cre mice.
Answer: In our study, our allele description is such that it is IL-1R1 and IL-1R3 is targeted, but because they are both transcript of the il1r1 gene which is the common gene targeted, we think that using IL-1R1-/- is appropriate.

European Journal of Immunology

- The number of worms in the caecum/proximal colon varies significantly between control animals in the different experiments (Figure 3A). Do the authors have any explanation?

Answer: We do not have an explanation for this difference, but we believe that this is due to unavoidable experimental variability.

- Page 5, line 22f: the authors might rewrite the sentence “ By positioning the loxP sites….”

Answer: We believe that this sentence is correct.

- Page 5, line 54: which genotype corresponds to “IL-1R1 fl/fl vav iCre+ homozygous mice”?

Answer: These mice are IL-1R1 fl/fl vav iCre+. We have deleted “homozygous”.

- Suppl. Figure 3: immature B cells might not be the major population in splenic IgM+, IgD- B cells (see figure legend).

Answer: We used this classification of B cells as a first approximation. We are aware that IgM+, IgD- B cell populations are a very complex population and also include for example recently activated B cells.

- Suppl. Figure 5 “cells” instead of “celss”.

Answer:

This has been corrected. Supplementary figures were inappropriately numbered, and Figure 5 should be labelled “Supplementary Figure 4 (Cont.)” This has been corrected accordingly.

<u>Second Editorial Decision – 12 November 2015</u>

Dear Dr. Pinteaux,

Thank you for submitting your revised manuscript ID eji.201546075.R1 entitled "Characterization of a conditional interleukin-1 receptor 1 mouse mutant using the Cre/LoxP system" to the European Journal of Immunology. Your manuscript has been re-reviewed and the comments of the referees are included at the bottom of this letter.

Unfortunately, Referee 2 was not satisfied with the revisions made and feels that the requested controls are necessary (first 2 points). The Editor-in-Chief agrees and we ask that you provide the requested controls. We apologise for any unclear wording at the last decision, which may have been interpreted that no new experimental data were necessary; we meant that no expansion of the scope of your story was

European Journal of Immunology

necessary. We hope that you will agree that it would be mutually beneficial to publish credible, controlled data, and we certainly would like to publish yours.

The journal does not encourage multiple rounds of revision and you should fully address the concerns of the referee in this final round of revision. Should you disagree with any of the referees' concerns, you should address this in your point-by-point response and provide solid scientific reasons for why you will not make the requested changes.

You should also pay close attention to the editorial comments included below. *In particular, please edit your figure legends to follow Journal standards as outlined in the editorial comments. Failure to do this will result in delays in the re-review process.*

Please note that submitting a revision of your manuscript does not guarantee eventual acceptance, and that your revision will be re-reviewed by the referees before a decision is rendered.

Once again, thank you for submitting your manuscript to European Journal of Immunology and we look forward to receiving your revision.

Yours sincerely,
Karen Chu

On behalf of Prof. Andreas Radbruch, with best regards to Prof. Müller

Dr. Karen Chu
Editorial Office
European Journal of Immunology
e-mail: ejied@wiley.com
www.eji-journal.eu

****

Reviewer: 2

Comments to the Author
In the revised manuscript of Abdulaal and colleagues my major concern regarding the functionality of the IL-1R1 fl/fl allele still remains as the authors do not analyze hematopoietic cells of mutant and C57BL/6 wt mice side-by-side.

European Journal of Immunology

Furthermore, the source of IL-1R1 expression in LPS-stimulated, IL-1R1-deficient hematopoietic cells is still obscure and thus prevents the interpretation of the presented results (as shown in Figure 2). The "intact" IL-1R1-dependent downstream signaling after LPS treatment of IL-1R1 deficient hematopoietic cells might be explained by the presence of IL-1R1 expressing hematopoietic cells that have not deleted the loxP flanked gene segment.

I do not understand the difference between "wild type corresponding to IL-22-/- " and "wild type to IL-1R1 fl/fl vav iCre (-)". What is the precise genotype of "wild type" animals analyzed in Figure 3? The authors should replace the misleading labeling of their graphs.

Minor Comments:
The sentence "By positioning the loxP sites around this exon, we prevented the production of the two known transcribed functional forms of the il1r1 gene." is incorrect as the positioning of loxP sites per se does not prevent the production of the transcribed functional forms, but cre mediated recombination at the loxP sites will then lead to the deletion and prevent the transcription of the functional forms.

Throughout supplementary figures 2 and 4 "florescence" should be replaced by "fluorescence".

In the reply to the editorial comments the authors state that their Western blot shows "a nonspecific band at a lower molecular weight that shows that similar amounts per sample were loaded." I am puzzled why this band in Fig. 2 is labeled as actin. Please clarify.

<u>Second revision – authors' response – 22 November 2015</u>

We thank the reviewer for the additional comments on our revised manuscript. We agree with the reviewer on all points raised, and have now included additional controls, and modified the presentation of some data in order to present a fully validated study more consistently. As a result, we now provide an amended version of the manuscript with highlighted changes, a final version of the manuscript, a new version of the main figures, and a new version of the supplementary materials.

Response to reviewer 2

Major concerns:
In the revised manuscript of Abdulaal and colleagues my major concern regarding the functionality of the IL-1R1 fl/fl allele still remains as the authors do not analyse hematopoietic cells of mutant and C57BL/6 wt mice side-by-side.

Answer: We fully agree with the reviewer regarding this very important point. We have now included additional control experiments (now presented in Figure 2A) to show that IL-1 responses are identical in C57BL/6 mice compared to IL-1R1fl/fl mice, demonstrating that il-1r1 gene is fully functional after insertion of LoxP sites. Furthermore, we show that IL-1 responses are functionally abrogated in our new IL-1R1-/- mice, demonstrating that deletion of exon 5 leads to complete inhibition of IL-1 signalling. In our new data, we found that IL-1-induced TNF□ release was non-significant and highly variable (which was already shown by our previous data that were presented in the previous version of our manuscript), and for this reason, we have decided to remove all TNF□ data. It is important to note that IL-1 responses in our new data (Figure 2A) are markedly lower than those found in our previous experiments (Figure 2B). This is due to the fact that those were independent experiments done with more than a year apart, carried out by different experimentalists and using different detection systems (ELISAs from BD Biosciences and R&D Systems).

Furthermore, the source of IL-1R1 expression in LPS-stimulated, IL-1R1-deficient hematopoietic cells is still obscure and thus prevents the interpretation of the presented results (as shown in Figure 2). The “intact” IL-1R1-dependent downstream signaling after LPS treatment of IL-1R1 deficient hematopoietic cells might be explained by the presence of IL-1R1 expressing hematopoietic cells that have not deleted the loxP flanked gene segment.
Answer: We agree with the reviewer that the source of IL-1R1 expression in LPS-stimulated cells remains uncharacterised and brings confusion for the interpretation of the results presented in Figure 2. We feel that the characterisation of cells that up-regulate the expression of IL-1R1 in response to LPS falls outside the remit of the paper and we have decided to remove all LPS treatments from Figure 1D. As suggested by the reviewer, the possibility that IL-1 signalling on IL-1R1-expressing hematopoetic and non-hematopoetic cells after LPS stimulation remains. To address this point, we have investigated whether endogenous IL-1 can contribute to LPS responses by treating cells with LPS in the presence of the IL-1 receptor antagonist (IL-1Ra). Our results found that LPS-induced TNF□, IL-6 and MCP-1 release was not significantly affected by IL-1Ra (NS vs. LPS, n=3), whilst IL-1Ra completely blocked IL-1 responses (* vs. IL-1□, n=2) (see table below), suggesting that IL-1 signalling is not involved critically in the cytokine release in the LPS response. We are providing those data for the reviewer’s consideration (see below).

	Control	LPS	LPS+IL-1Ra	IL-1β	IL-1β+IL-1Ra	IL-1Ra
TNFα	10.7±1.4	340.7±41.4	336.5±58.6^{NS}	25.1±10.1	8.6±1.1*	14.5±3.9
IL-6	17.3±8.8	3062.8±484.4	2544.5±256.0 ^{NS}	31.0±13.9	3.3±2.2*	6.4±3.2
MCP-1	3.5±0.9	20.2±3.9	20.6±4.9^{NS}	16.1±2.6	5.9±2.2*	2.1±0.9

I do not understand the difference between “wild type corresponding to IL-22-/- “ and “wild type to IL-1R1 fl/fl vav iCre (-)”. What is the precise genotype of “wild type” animals analyzed in Figure 3? The authors should replace the misleading labeling of their graphs.

Answer: As stated previously, wild type have been used as corresponding controls to IL-22-/-, therefore the genotype is C57BL/6 wild type mice. In this experiment presented in Figure 3, which included many experimental genotypic groups, we have used IL-1R1 fl/fl vav iCre (-) as littermate controls to IL-1R1 fl/fl vav iCre (+). We have deliberately avoided to include C57BL/6 wild type controls to IL-1R1 fl/fl vav iCre (-) because worm burden was identical between between IL-1R1fl/fl and wild type mice (Figure 3A and B), but we also now provide additional evidence in Figure 2A that il-1r1 gene functionality is not affected by insertion of LoxP sequences in IL-1R1fl/fl mice.

Minor Comments:
The sentence “By positioning the loxP sites around this exon, we prevented the production of the two known transcribed functional forms of the il1r1 gene.” is incorrect as the positioning of loxP sites per se does not prevent the production of the transcribed functional forms, but cre mediated recombination at the loxP sites will then lead to the deletion and prevent the transcription of the functional forms.
Answer: We agree with the reviewer and have now amended the sentence to reflect that deletion is induced by Cre recombinase.

Throughout supplementary figures 2 and 4 “florescence” should be replaced by “fluorescence”.
Answer: This has now been corrected in all supplementary figures.

In the reply to the editorial comments the authors state that their Western blot shows “a nonspecific band at a lower molecular weight that shows that similar amounts per sample were loaded.” I am puzzled why this band in Fig. 2 is labeled as actin. Please clarify.
Answer: This is a result of a sloppy use of language made in our reply to the editorial comments, and we confirm that the lower band shown in figure 1 is indeed □-actin. (Figure and figure legend labelling appropriately).

Third Editorial Decision – 10 December 2015

Dear Dr. Pinteaux,

It is a pleasure to provisionally accept your manuscript entitled "Characterization of a conditional interleukin-1 receptor 1 mouse mutant using the Cre/LoxP system" for publication in the European Journal of Immunology. For final acceptance, please follow the instructions below and return the requested items as soon as possible as we cannot process your manuscript further until all items listed below are dealt with.

Peer review correspondence

Please note that EJI articles are now published online a few days after final acceptance (see Accepted Articles: http://onlinelibrary.wiley.com/journal/10.1002/(ISSN)1521-4141/accepted). The files used for the Accepted Articles are the final files and information supplied by you in Manuscript Central. You should therefore check that all the information (including author names) is correct as changes will NOT be permitted until the proofs stage.

We look forward to hearing from you and thank you for submitting your manuscript to the European Journal of Immunology.

Yours sincerely,
Karen Chu

on behalf of Prof. Andreas Radbruch

Dr. Karen Chu
Editorial Office
European Journal of Immunology
e-mail: ejied@wiley.com
www.eji-journal.eu
